# A Renewed Call to Safeguard Public Health Epistemology

**DOI:** 10.3389/ijph.2021.1604009

**Published:** 2021-05-05

**Authors:** Joshua S. Yudkin

**Affiliations:** University of Texas Health Science Center at Houston, Houston, TX, United States

**Keywords:** public health, epistemology, transdisciplinary, methodology, research

**The IJPH series “Young Researcher Editorial” is a training project of the Swiss School of Public Health**

Human beings are born to ask “why.” We ask the question to satisfy the fundamental human quest for knowledge, and this question lies at the heart of science. Science is a systematic approach to validate truth, and in that system, numbers and words are the tools we use to communicate. With these tools, we attempt to root abstract concepts in reality, and they can help us exchange ideas effectively and accurately. But scientific tools are often misused. For example, public health scientists can lose their perspective and focus on ephemeral and personal priorities like advancing their careers instead of the communal goals that increase our collective knowledge and improve our collective health and wellness. As producers and consumers of public health’s epistemic framework [1], we are obligated to continually assess and ensure integrity in our quest for knowledge.

We have a moral and professional obligation to provide sufficient information to other researchers so they can replicate our work. Replicability is critical to determining causality [2] and ensuring validity [3]. Too often, authors do not adhere to best practices and reporting guidelines [4]. In major epidemiological journals, 25–39% of studies do not include sufficient methodological detail to allow others to replicate their results [5]. Moreover, only 28% of published research sufficiently base their methodology—such as variable selection—on recommendations from existing research or their collected data. As evidenced, the majority of research is neither contextualized sufficiently nor aligned with best reporting practice [5, 6].

Our collective public health goals and individual professional goals may come into conflict. As public health scientists, we are dedicated to preventing disease and promoting health [7]. But too often, the quality of research is sacrificed in the pursuit of the high quantity of publications needed by employers to continue in this important work. The misalignment between our scientific goals and professional constraints delays and detracts from achieving our shared aforementioned goals of increasing knowledge and improving collective health and wellness and sometimes creates waste and encourages unethical practices [8]. In fact, only a fraction of research is ever implemented into practice, 17 years later [9].

Moreover, not all of the information published is valid. In spite of guidelines and extensive discussion on effective reporting and identifying excellent research, noise is still prosumed—both produced and used in research endeavors [10]. As a result, public health scientists need to continually assess the evidence through independent evaluation of the source, even when it is drawn from peer-reviewed literature, before they use it in their research and publications. The amount of noise and misinformation is rapidly increasing and multifactorial: predatory low-quality scientific journals, professional pressures to publish high quantities of manuscripts, and the exaggerated and uncontextualized dissemination of findings to the public are notable causes.

There are three immediate opportunities to help safeguard public health epistemology and improve health outcomes. First, transdisciplinary collaborations can improve dialogue and consistency across all peer-reviewed scientific publications. Additionally, they lead to better reported innovative evidence-based methodologies for their diverse stakeholders, ultimately improving both validity and replicability. Finally, transdisciplinary collaborations can improve our translation speed and increase our collective impact, quicker. Good examples of such collaborations currently exist at the intersection of public health and behavioral economics [6].

Second, we can also incentivize research quality over research quantity. As new data sources and types emerge, we can re-imagine how we define variables to increase the validity of our results. For example, though it is common in the literature, it may no longer be appropriate or accurate to use English proficiency as a proxy for acculturation in studies of U.S. immigrants. Though previously validated tools may be easy to find and use, they may lose their relevance and accuracy. Given that more accurate measurement instruments may be available, the continued use of a less accurate tool, albeit validated, is a disservice to the field.

Finally, as producers and consumers of knowledge, we can systemically incentivize and individually choose to engage with quality research in order to advance the field with integrity and follow established best practices and guidelines. Specifically, researchers should articulate and follow a priori hypotheses and procedures and ensure they present their research in sufficient methodological detail so others can replicate it. Additionally, researchers should locate their work within the published literature on their topic and publish their full research, regardless of outcome, as quickly as possible in order to reduce the translation lag from research to practice and improve health outcomes quicker.

To safeguard public health epistemology, we must individually choose and collectively encourage scientists to engage in rigorous research methods guided by established best practices. Science, an aspect of the human search for truth, is the systematic approach to authenticating and validating information. Each decision—from building our research teams to the variables we include in a regression model—should be intentional, evidence-based, and aligned with scientific best practices. This text is a renewed call for the field of public health that highlights current concerns and opportunities in our production and consumption of knowledge.
